# Design and Testing of a Multi-Sensor Pedestrian Location and Navigation Platform

**DOI:** 10.3390/s120303720

**Published:** 2012-03-19

**Authors:** Aiden Morrison, Valérie Renaudin, Jared B. Bancroft, Gérard Lachapelle

**Affiliations:** PLAN Group, Schulich School of Engineering, The University of Calgary, 2500 University Drive NW, Calgary AB, T2N 1N4, Canada; E-Mails: ajmorris@ucalgary.ca (A.M.); renaudin.valerie@gmail.com (V.R.); j.bancroft@ucalgary.ca (J.B.B.)

**Keywords:** GPS (Global Positioning System), GNSS (Global Navigation Satellite System), INS (Inertial Navigation System), IMU (Inertial Measurement Unit), barometer, magnetometer, high sensitivity, pedestrian navigation, indoor navigation, sensor fusion

## Abstract

Navigation and location technologies are continually advancing, allowing ever higher accuracies and operation under ever more challenging conditions. The development of such technologies requires the rapid evaluation of a large number of sensors and related utilization strategies. The integration of Global Navigation Satellite Systems (GNSSs) such as the Global Positioning System (GPS) with accelerometers, gyros, barometers, magnetometers and other sensors is allowing for novel applications, but is hindered by the difficulties to test and compare integrated solutions using multiple sensor sets. In order to achieve compatibility and flexibility in terms of multiple sensors, an advanced adaptable platform is required. This paper describes the design and testing of the NavCube, a multi-sensor navigation, location and timing platform. The system provides a research tool for pedestrian navigation, location and body motion analysis in an unobtrusive form factor that enables *in situ* data collections with minimal gait and posture impact. Testing and examples of applications of the NavCube are provided.

## 1. Introduction

The development of the NavCube multi-sensor navigation platform is primarily motivated by: (i) the desire to accurately position and navigate pedestrians and objects in urban and indoor environments, (ii) simultaneously collect various sensor data at various points on the body, (iii) being unobtrusive when mounted on the body and (iv) unpretentiously collect data as though an individual was not configured with a data collection system (*i.e.*, no physical change in the gait or posture). A system meeting these criteria is capable of providing extensive data *in situ* with the ability to compare various processing methods as a function of different sensor combinations or sensor locations (e.g., an ankle mounted tracking device *vs*. a smart phone).

While superficially comparable to other classes of navigation applications, pedestrian navigation brings numerous challenges. An important challenge is cost, as the navigation system used by a pedestrian will generally be a component of another unrelated system such as a communication system or smart phone. Often the price of the components used in the navigation system is to be minimized while factoring in weight, size and power demands placed upon the host device.

An additional challenge to practical pedestrian navigation systems involves the large range of dynamics that may be produced by human motions, which may exceed the measurement ranges of inertial navigation sensors [1]. As a consequence, a pedestrian navigation system must either omit inertial measurement units (IMUs) or alternatively select components, which are either capable of measuring the full range of expected human dynamics or implements algorithms which do not require continuous unsaturated observation of the user’s motion.

Another consideration is the fact that a pedestrian will operate in environments that either limit or deny availability of satellite navigation signals such as those from GPS. While GNSS signals are extremely useful outside, their weak power levels and signal reflections limit their use in many indoor settings. As such, a pedestrian navigation platform should be capable of prolonged operation in environments where satellite navigation signals are either unavailable or only intermittently accessible. When operating under these conditions, a navigation system must fall back on other positioning methods, or include complementary relative positioning systems, which allow short term propagation of a known position and heading.

Much research has been conducted in attempting to improve inertial based pedestrian navigation accuracy indoors (e.g., [2–16]), but very little is dedicated to methods of improving the data collection process’ within the study. It is therefore the scope of this paper to address a data collection tool that will facilitate an increased rate at which researchers can study pedestrian navigation and extend applications to the biomedical field. For examples of biomedical and navigation fields converging see [17–21]. Magnetometers are also being used to improve accuracy through innovative algorithms (e.g., [22–24]).

To permit indoor operation of the NavCube, complimentary relative positioning and navigation sensors such as IMUs, magnetometers, and barometers are included in the system design along with provision for future use of non-GNSS absolute positioning methods, such as received signal strength indicators (RSSI) from 802.11 network based positioning systems and wheel speed sensors.

The NavCube functionalities can be used in other applications, scenarios and research. Biomechanics, bioengineering, guidance, robotics and animation are all engineering fields that used to be independent from each other but now have merging sensors and principles. Indeed, with the progress in miniaturizing sensors, the data of interest for a specific domain is now available to others at little or no additional cost. For example, sport watches can include a GPS receiver and a heart rate monitor [25]. Accelerometers, gyroscopes, magnetometers and barometers are embedded in smartphones. The hardware is used in daily life activities and provides continuous information about their users. This is stimulating research in developing new processing methods for improving living conditions. Consequently, several new measurement units have been designed for conducting research on multiple aspects of micro-electromechanical measurement systems (MEMS) data processing for physiological or industrial applications and the combination of different scientific competences lead to novel results.

A major evolution in the usage of mobile measurement units comes from the fact that until recently they were rigidly attached to vehicles for navigation purposes whereas nowadays they are carried by human of all ages in their hands, pockets or bags. Globally two categories of measurement units exist. The first one consists in systems able of recording measurements in a specific location and often equip a dedicated room. They are essentially capturing human movements or used for shaping industrial components. The second category consists of IMUs whose use is not restricted to a specific area and can be carried out by the user in indoor and outdoor environments. Although not exhaustive, a survey of tracking systems for biomedical analysis (e.g., human movement and stroke rehabilitation) can be found in [26].

Among the first category are optical tracking systems that calculate the position and orientation of markers rigidly attached to the body, which are readily available in laboratories (e.g., [27–29]). They often use real-time digital photogrammetry and optical triangulation techniques to track the markers. A classical biomechanical application of these systems is the measurement of the exact nature of human body segments during motion. Completed with laser scanning or marker free-technology [30], the measurements, which are constrained in a specific volume, can also be used for industrial metrology (e.g., 3D modeling of automotive parts). Unfortunately, constraining the activity or body of interest to a specific location and area limits the applications range. Thus IMUs, members of the second category, are principally tri-axis sensors integrated on a single main circuit board, which has its own power management and data logging system. The MPU-6000/6050 product family of tri-axis accelerometer and gyroscope from Invense [31], the tactical grade ten degrees of freedom inertial sensor ADIS16375 from Analog Devices [32] or the iNErtial MOdule V2 from STMicroelectronics [33] are examples of these IMUs. Some more advanced solutions comprise a logging system for recording time synchronized data from multiple IMUs attached to the body along a distributed architecture. Widely used in Europe, the products based on inertial sensors proposed by Xsens are recording synchronized data from multiple IMUs. They provide different options for mounting the sensors on the user’s body and are principally oriented toward the use of accelerometers and gyroscopes, especially with the six degrees of freedom MTx IMU [34]. Solutions for using magnetic field data and GPS signals are also available.

The NavCube presented herein provides the ability to compare different GNSS receivers thus allowing comparison of different antenna combinations, locations and receiver type in pedestrian navigation activities. This benefit is in response to the advent of new GNSS signals and the wish to use GNSS pseudoranges and Doppler in signal degraded environments. As a consequence, research in navigation based on multiple IMUs (e.g., [35,36]) and GNSS has to follow the latest developments for integrating them in successful navigation solutions.

In order to develop algorithms for fusing inertial and GNSS signals for pedestrian navigation applications, especially using high sensitivity receiver (HSGPS) the NavCube comprises up to 11 sensor sets (IMUs, barometers and magnetometers), a multi-frequency survey grade GNSS receiver, two HSGPS receivers, and a high sensitivity GNSS (HSGNSS) receiver. In the following two sections, the design of the NavCube hardware platform will be described, and applications examples are provided which are common challenges facing pedestrian navigation research.

## 2. System Design

**Figure 1.**
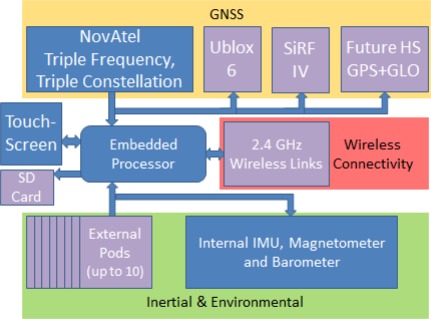
Block diagram of NavCube platform. Core components are shown in blue, modular devices shown in violet including HSGPS and HSGNSS receivers. Currently the system is configured to utilize dual u-blox 6 HSGPS receivers rather than the depicted combination of u-blox and SiRF based receivers.

The NavCube has been designed to carry a wide variety of navigation sensors including accelerometers, gyroscopes, barometric pressure sensors, magnetic field sensors and future sensors including wheel speed sensors. Modularity has been emphasized where possible to allow the study of a variety of cutting edge sensors, with the option to exchange these as future developments in sensor technology occur. The primary strategy for obtaining this modularity was the subdivision of the system into core components and peripheral sensors. The block diagram of Figure 1 shows the modular/external elements of the system in violet, including the connection points for up to ten wired external sensors.

In order to support any selection of current and future sensors for any given testing scenario, external pod connections are provided by the NavCube to allow up to 10 external sensors to be interfaced to, synchronized with, and both powered and logged by the NavCube. This approach allows the replacement of any external and several internal systems when future versions become available, or to allow the side by side evaluation of past and present technologies. The multitude of external sensors supported allows multi-IMU pedestrian navigation applications such as those discussed in [35,36] as well as to provide an evolution path for the system to add new sensor types in the future. Combined with the Adaptable Sensor Pod (ASP) developed internally, which contains inertial barometric and magnetic field sensors, the NavCube is capable of instrumenting a pedestrian to provide this combination of data from 11 or more locations on the body simultaneously.

**Figure 2.**
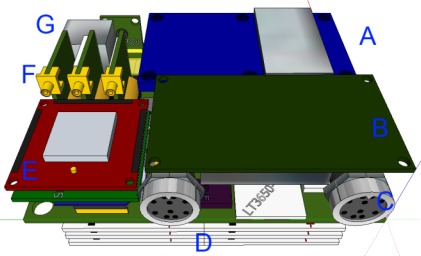
NavCube internal component sketch. Components are as follows—Element **A**: GPS, GLONASS L1/L2 100 Hz satellite navigation receiver and timing reference. Element **B**: Internal IMU with Magnetometer and Barometer. Element **C**: External Pod connection port. Element **D**: System batteries. Element **E**: 2.4 GHz Wireless modules. Element **F**: High Sensitivity GPS and GPS+GLONASS riser boards. Element **G**: Other internal sensors.

To facilitate the use of the system in typical pedestrian navigation applications the weight, size and physical dimensions were necessarily constrained, while device power storage and conditioning was integrated into the system enclosure in such a way as to provide power for both internal and external sensor systems. The layout of internal components was optimized to reduce system volume, as shown in Figure 2.

**Figure 3.**
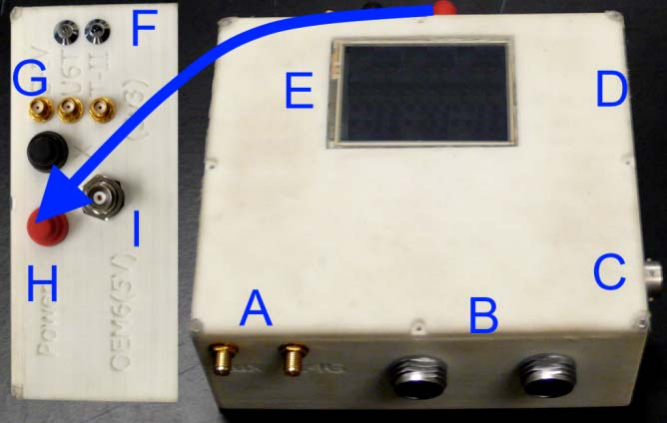
NavCube system assembled in enclosure. Features are as follows—Element **A**: 2.4 GHz RF link antenna connectors. Element **B**: External sensor pod data, power and timing connectors. Element **C**: Charge connector. Element **D**: SD card socket (side of enclosure). Element **E**: Touchscreen. Element **F**: Indicator lights. Element **G**: High-Sensitivity GPS/GNSS receiver antenna connections. Element **H**: Control buttons. Element **I**: GPS, GLONASS multi-frequency receiver antenna connection.

Not shown in Figure 2 is the removable storage media socket situated between the main circuit board and component A. All data collected by the system is internally synchronized and logged to this removable storage media for post processing. Element B in Figure 2 is identical to the ASP inertial, barometric and magnetic sensor ensemble. Figure 3 shows an external view of the assembled NavCube system installed within a custom laser deposition printed enclosure.

The internal sensors of the NavCube can be separated into the GNSS and non-GNSS categories, with the former containing the high sensitivity and high precision GPS and GLONASS receivers, and the latter containing the accelerometer and gyro triads, barometer and magnetometer. For comparison, the former category is enumerated in Table 1.

The list of GNSS receivers in Table 1 is not an exhaustive list of all possible supported GNSS systems, but rather indicates the receiver systems for which support boards have been designed to date. The OEM628 receiver forms the basis of the internal timing control of the NavCube and serves as the master timing reference for the rest of the system in addition to providing a GNSS receiver capable of very accurate phase measurements for Real-Time Kinematic positioning and high update rates. Secondary GNSS modules are installed via daughter cards, which allow future upgrade and replacement of these receivers, or the installation of multiple instances of the same receiver module when desired. Currently the system is operated with dual u-blox 6T modules, omitting the SiRF IV in order to gain multiple 5 Hz high sensitivity GPS sources.

Following the same design philosophy for non-GNSS sensors within the NavCube as that followed for the secondary GNSS sensors, the non-GNSS sensors are installed on a replaceable daughter card to permit future upgrade as more advanced models become available. The design cycle of this component was shortened substantially by sharing a common electrical and similar physical design to the sensor boards contained in the ASP pods. Currently the non-GNSS carrier board mounts the sensors indicated in Table 2, though it is intended to upgrade these at a future date as part of the system evolution. The ASP communication is via RS-232 signaling protocol, EIA-232 voltage levels, at 115,200 baud rate.

The ADIS16375 MEMS IMU is noteworthy for being a sub $1,000 inertial navigation solution, which is sufficiently stable in terms of gyro bias stability to detect the rotation rate of the earth under ideal conditions. Earth rotation detection is typically a task that requires the use of a much higher priced, much higher power consumption, and often much larger and heavier Fibre Optic Gyro (FOG) or Ring Laser Gyroscope (RLG) based IMU. While the ADIS16375 is still far less stable than FOG and RLG solutions, for a pedestrian navigation system designed to be effortlessly carried on the person of the user, the 100 gram mass of the ADIS16375 IMU is an excellent compromise of size and weight for performance.

To maintain the low weight of the system while gaining the capability of determining heading autonomously, the Honeywell HMC5883L magnetometer was selected for integration into the NavCube and ASP pods as a sub component of the inertial sensor carrier board. With a mass of less than 1 gram and negligible power consumption relative to the IMU, the inclusion of an HMC5883L enhances utility without degrading usability. Similarly, by integrating the Bosh Sensortec BMP085 barometer, the system is able to estimate changes in its elevation over time. Though sensitive to changes in weather pattern, as well as to the pressurization level of building ventilation systems, the barometer remains a useful tool in the estimation and constraint of vertical motion.

The NavCube weighs 1.2 kg, while each ASP is 0.1 kg. The total weight of the system including the NavCube, four ASPs, the wiring harness, a survey grade mobile GNSS antenna and its antenna cable, 3 low cost mobile antennas and a custom neoprene case is 2.7 kg.

### 2.1. Time Synchronization

Most navigation processing software assumes that each sensor is synchronized with GPS time (or another atomic time scale such as UTC where offsets are known). Thus, timing within the NavCube requires each sensor’s output to be either time-tagged or synchronized with its internal clock via a Pulse Per Second (PPS) signal. The OEM628 GNSS receiver provides a PPS signal that is distributed to each ancillary module to maintain timing.

**Figure 4.**
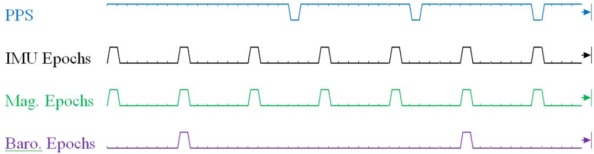
ASP PPS and Sensor Epoch Alignment Example.

Within the ASP, the IMU, magnetometer and barometer timing is driven by a custom timing and data controller. This controller ensures that the 100 Hz IMU, 100 Hz magnetometer and 25 Hz barometer data has a near zero delay between sensors. Additionally, the controller steers the sampling train of all sensors such that the epochs of the IMU and barometer coincide with the next predicted PPS event. As shown below in Figure 4, the steering process takes 2–3 s to complete, and ensures that users with multiple ASP units driven by a UTC synchronized PPS train will have simultaneous IMU and magnetometer samples within ±100 μs. Barometric pressure sample times are similarly steered to maintain their alignment to IMU/magnetometer sample epochs. However, as is depicted in the timing diagram below, the epoch of barometric sensor output is not steered into alignment with expected PPS events.

The timing sequence of each ASP has three key aspects. First, the ASP time-stamps all events in terms of an internal free-running 32-bit counter. Naturally this counter will overflow back to zero on the clock cycle following 232-1. As such the user must take this overflow into account when handling data timing. Second, in order to unambiguously map the ASP PPS received epochs to UTC/GPS time, it is necessary to control the reception of the first time pulse and to know when this pulse occurred (e.g., by using the EVENT input from the NovAtel receiver log). Third, the timestamp data of the ADIS16375 sensor is compensated for the effects of digital filtering lag within the IMU. The implemented filter has a step response with a delay of 20 ms from the time of actual physical excitation. For this reason IMU timestamps will appear to refer to ‘the past’ with respect to other sensors, despite the fact that the actual sampling was simultaneous. When implementing a navigation filter which will utilize inertial data as well as barometric or magnetic data this timing behavior is required to be appropriately handled, as it should not be assumed that the samples of each sensor within the same frame are referred to the same moment in time.

### 2.2. Autonomous Operation

The NavCube operates on a four cell 88.8 Watt-hour lithium polymer battery pack. Given that the batteries data sheets are typically overly optimistic in terms of rated energy the available energy is approximately 82.1 Watt-hours within the batteries. A battery protection system ensures that the batteries remain within operating limits when nearing low power. Two voltage rails (3.3 V and 5.0 V) were required and thus power conversion circuitry was required. This reduced the available power by approximately 14%, to about 70.6 Watt-hours. Table 3 provides the net power usage of each component within the NavCube. The OEM6 GNSS receiver consumes the most power typically at 1.8 W, while each sensor pod requires 1.0 W. With a full complement eight sensor pods, the run time is approximately 4.5 h.

The NavCube file system controller supports the use of SD, and SDHC memory cards. Use of cards with a 133x+ or class 10 rating is best, but UHS 1 or 2 rated cards also work. Both FAT16 and FAT32 file systems are compatible with the system. Under these file systems maximum file size limits of two GB and four GB exist, respectively. Data collected exceeding these limits are stored in file sets and manually appended post mission. Data rates for each GNSS sensor are selectable by the user. Table 4 provides the approximate data rates of the maximum frequency of the observations. Actual data storage (and power draw) depends on many factors including satellite availability and the surrounding conditions/environment. The sensor pods however are more stable in their data rates.

## 3. Applications and Field Testing

In this section field tests carried out with the NavCube and ASP pods are described to showcase its versatility in navigation based research. The first of these is a series of testing scenarios focused on the use of embedded sensors in handheld device navigation.

### 3.1. Handheld Navigation

With the proliferation of smart phone devices which provide enhanced functionality given accurate location awareness, new methods of navigation and positioning indoors where GNSS is not available are needed. Step detection and step length estimation algorithms that function reliably within handheld devices, both during natural motion, as well as that which would be expected if the user were typing on a smartphone or otherwise interacting with a smartphone, rather than simply holding it on the side, are being pursued.

**Figure 5.**
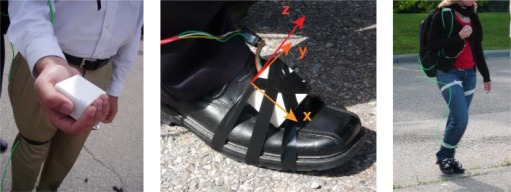
Pedestrian wearing the NavCube in a backpack with one IMU fixed on each foot and one IMU held in hand.

Five men and five women participated in a 450 m flat test course data collection for conducting gait analysis of pedestrians walking, but only using a handheld inertial sensor. Algorithms for detecting steps, classifying the user’s activity and evaluating step frequency had been developed previously (e.g., [37]). To confirm that the proposed step detection methods based on handheld device were reliably detecting user footfalls in both texting and normal walking modes of use, the NavCube was configured to include a foot mounted IMU. A shown in Figure 5, by placing one ASP on each foot as a step detection reference, in addition to one ASP unit in hand and one in the waist mounted NavCube, the effectiveness of step detection through handheld inertial sensors was directly observable.

**Figure 6.**
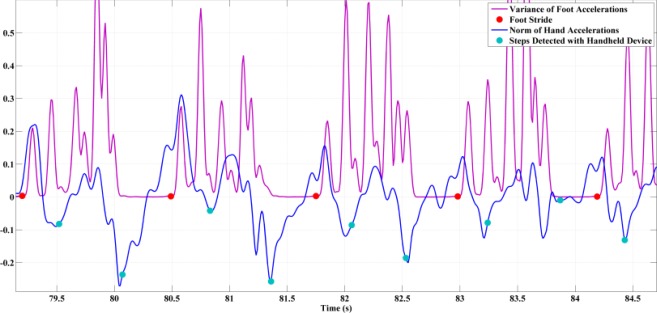
Comparison of the steps detected with the handheld inertial sensors (cyan dots) and the strides events detected with the sensors rigidly attached to the left foot (red dots).

The handheld IMU senses the footfalls of both feet, whereas a foot mounted IMU senses only the beginning of the foot corresponding to the stride. Figure 6 presents the results of the step detection process and effectively observes this phenomenon since for one footfall, marked with a red dot, and two steps marked with the cyan dots, detection occurs with handheld inertial sensors.

**Figure 7.**
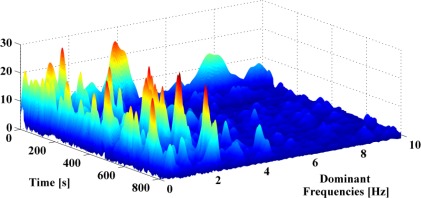
Time series of the spectrum of user hand motion when walking naturally with a handheld device. Dominant frequencies are observed in the range 0–5 Hertz and can be used to classify the motions of the hand and user.

Figure 7, is also useful when attempting to categorize whether the motion being measured is the result of the handheld device swinging with the users stride or if it is indicative of a fixed body-device orientation, which is expected during interaction of the user with the device or if it captures a hand motion that is considered as parasite for the navigation process. Since different navigation algorithms can be tailored to each of these user motion categories, reliably differentiating between them improves the navigation performance achievable with handheld devices. Data analysis and results of these handheld navigation tests are presented in [37].

### 3.2. Pedestrian Navigation

**Figure 8.**
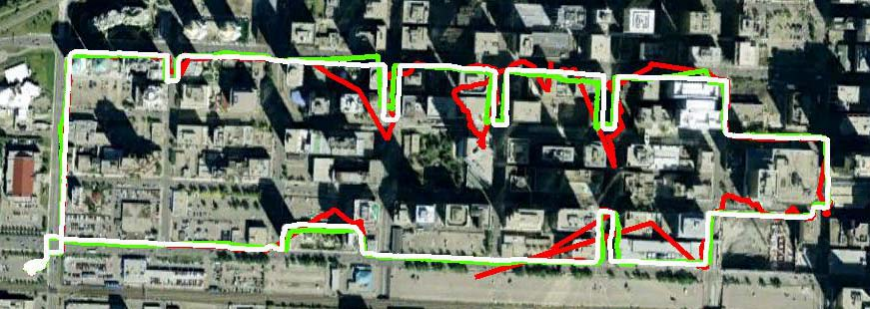
Pedestrian testing results collected using the NavCube in the dense urban canyon environment of downtown Calgary. The white trace is the truth trajectory produced by a SPAN™ LCI tactical grade inertial navigation system (INS), the red trace is the trajectory produced by a GPS+GLONASS GNSS receiver and the green trace is derived from combined GPS+GLONASS with IMU, barometric, and magnetic field measurements [38].

Some applications allow or require that the navigation system or tracking device be mounted on the user’s ankle. Using the NavCube as a platform, data was collected from an ankle-mounted combination of ASP pod and the antenna from the NavCube integrated u-blox 6 HSGNSS system. The results of this collection are shown in Figure 8 where data from the reference trajectory obtained from a SPAN™ LCI tactical grade inertial system is shown in white, while HSGPS alone is in red, and the combined ASP plus HSGPS ankle data is shown in green. The algorithms and additional analysis are provided in [38].

The data from the ASP and HSGPS receiver were processed using the Multiple IMU Navigation Software (MINS), originally developed in [39] and since adapted to integrate barometric measurements and Quasi Static Field (QSF) technology introduced in [40]. While originally developed for applications utilizing multiple IMUs, the MINS software package is now capable of processing navigation data from multiple sensor types. In addition to GPS INS integration, the MINS software package is capable of utilizing barometric pressure sensors to derive height information, as well as updating the heading and gyro bias estimates of the INS by using the QSF updates from a magnetometer.

In an urban canyon navigation scenario, the MINS software estimates the biases in the IMU gyroscopes and accelerometers when GNSS is available, and subsequently uses these estimated corrections to determine the position and heading of the user via the IMU when GNSS data is unavailable or degraded. The addition of QSF allows the MINS software to estimate gyro biases when the system passes through a region of stable magnetic field, which constrains the system heading error over time, allowing more accurate position estimates in GNSS degraded environments. Simultaneously, MINS may make use of barometric pressure data to contain errors in the navigation solutions vertical component. The aggregate result of MINS processing of the input of a GNSS sensor combined with an ASP pod mounted on the user ankle with a calibrated magnetometer per [41], and a barometer is a considerable improvement in position accuracy relative to a GNSS only solution when navigating in an urban canyon.

### 3.3. Sport Applications

**Figure 9.**
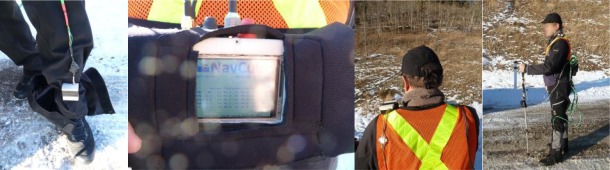
Hiker wearing the NavCube at the waist, two IMUs attached to the leg above the boots, one IMU on the shoulder and a GNSS antenna on the other shoulder.

Navigation and body limb motion monitoring are becomingly more critical in athletic training and coaching. Numerous sports, including skiing, running, rowing and field hockey are using multisensory systems to track and improve performance (e.g., [26,42–46]). Because a large part of sport activities takes place outdoors, where users are free to perform any kind of motion, equipment used for performance evaluation must be light and noninvasive. Data collected by a hiker wearing the NavCube has been used for assessing different navigation and biometric parameters. Two APSs were rigidly attached to the legs above the hiking boots and another on the left shoulder; the core module of the NavCube was carried at the waist using a neoprene case and belt, two antennas located on the right shoulder and one antenna rigidly connected to the ASP located on the left foot. The complete data collection setup is illustrated in Figure 9.

**Figure 10.**
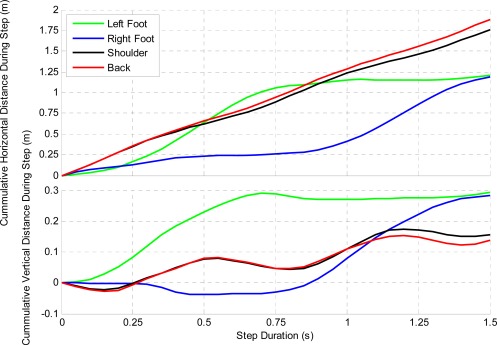
Hiker’s Average Displacement at Various Sensor Locations during each Step.

**Figure 11.**
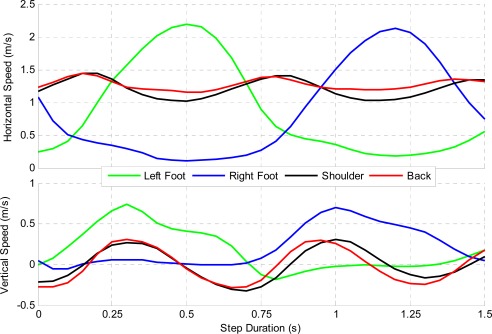
Hiker’s Average Velocity at Various Sensor Locations during each Step.

The hiker hiked 3.5 km and ascended 700 m vertically in 68 min and took approximately 2,750 steps (one step is from heel lift to heel lift of the same foot). Each step length and velocity was averaged to determine a nominal step profile and is shown in Figures 10 and 11. The left foot was used to determine the start and end of each step. Figure 10 shows the displacement of each ASP, both horizontally and vertically. Each profile varies slightly due to uncorrectable errors, but these errors are limited to a few centimetres. It can be derived from this figure that the hiker has an average step height of 29 and 30 cm and a step length of 119 and 121 cm for the left and right feet while hiking. The back and shoulder sensors are in close agreement, but vary likely due to the user bending forward at steeper inclines.

Figure 11 provides a profile of the speed during the single gait cycle. The user had an average maximum foot speed of 2.20 m/s and 2.13 m/s for the left and right feet, respectively. The shoulder and back velocities also provide a clear view of the periodic speed related to each step. The vertical speed indicates a rising and falling velocity during each step on the order of 0.25 m/s. A GPS only, epoch-by-epoch least-squares solution averaged over the same intervals confirmed the negative velocities. This results from the hiker leaning forward at each step.

### 3.4. Use of Magnetometers for Navigation

The Earth magnetic field is a force that can be sensed everywhere and used to compute azimuth. If the magnetometer is in hand, there exists an ambiguity of the sensor’s orientation in its body frame and the user’s direction of travel. Thus multiple sensors are often required to determine the hand’s azimuth and the azimuth of the user to assess algorithms using only one set of sensors.

An important condition for using magnetometers is that the sensor should measure only the earth magnetic field and no other artificial magnetic source. Consequently any field produced by electronic components embedded in the device has to be mitigated with proper calibration. These fields produce a magnetic deviation consisting of hard and soft iron perturbations. Hard iron effects produce a permanent magnetic deviation resulting from magnetized materials or other fixed magnetic field sources within the vicinity of the magnetometer. Soft iron effects correspond to induced magnetization caused by the permeability of ferromagnetic compounds onboard the sensor and that are interacting with an external field. A calibration method for mitigating the magnetic deviation but also all errors due to fabrication issues developed previously [41,47] was applied to the hiking data.

**Figure 12.**
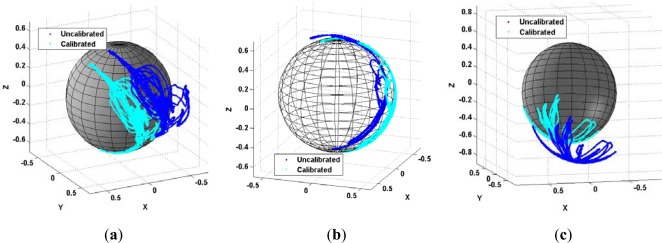
Biased and distorted magnetic field observations (blue) are calibrated resulting in the nearly ideal observation sphere (cyan) for the magnetometers embedded in IMUs attached to the left foot (**a**), the right foot (**b**) and the shoulder (**c**).

**Figure 13.**
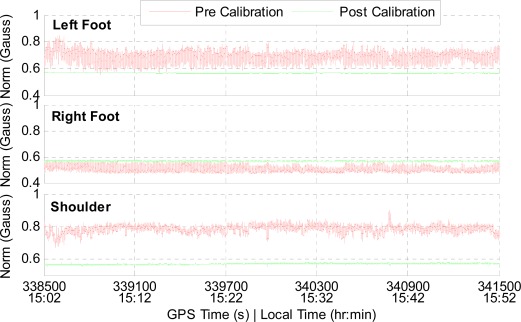
Norm, before (red) and after the calibration (green), of the magnetometer’s field measured with the IMUs attached to the left foot, the right foot and the shoulder. The norm of calibrated data equals the norm of the local Earth Magnetic Field: 0.57 Gauss for this dataset and location.

Using the NavCube, it is possible to compare the calibration results of different magnetometers mounted on body. In Figure 12, calibrated and non-calibrated magnetic field measurements are shown for the left foot (a), the right foot (b) and the shoulder (c), respectively. When visualized in the vector space, ideal magnetic field measurements shape a perfectly spherical representation of a constant local field centered at the origin, however due to errors, the surface is an ellipsoid not centered at the origin. As it can be observed in Figure 12, even with all sensors carried by the same hiker, the perturbation effect is unique for each magnetometer. This is due to individual fabrication issues but more specifically to surrounding ferromagnetic compounds, for example metallic parts in the shoes. In Figure 13, a comparison of the norm of the measured local magnetic field magnitude in a calibrated (green) *versus* un-calibrated (red) magnetometer is given. Although the raw data is totally different for the three IMUs, the apparent field magnitude fluctuations are removed for all sensors leading to the norm of the Earth magnetic field extracted from the International Geomagnetic Reference Field (IGRF).

Application of these corrections allows the estimation of accurate headings despite the local environment of the magnetometer containing a plethora of active electronics and some ferromagnetic material. The new algorithms used above are discussed in [41] while the use of similarly calibrated low cost amorphous magneto-resistive (AMR) sensors for navigation in magnetically perturbed indoor environments are discussed in [47].

## 4. Conclusions and Future Plans

The system developed herein has proved an invaluable pedestrian navigation system due to its capability to power, synchronize, log and support a plurality of pedestrian navigation sensors. With applications of the system already spanning pedestrian navigation research topics, planned extension to include additional sensors will further broaden its range. The system also provides valuable information for biomechanics and sport applications.

Future sensors under consideration include supplementary absolute positioning via 802.11 wireless network observations through an internal network card, as well as the evolution of relative positioning capabilities through periodic replacement of both internal and external IMUs. The next planned non-GNSS enhancement is expected to be activation of the capability of the system to interface to a tactical grade IMU in order to provide researchers with a high accuracy indoor reference trajectory.

## Figures and Tables

**Table 1.** GNSS receivers supported by the NavCube system and their typical application type.

**Receiver**	**Application**	**Signals Supported**	**Update Rate**
OEM628	Survey, Machine Control, Timing, Real-Time Kinematic Positioning	L1/L2 GPS/GLONASS (L5 with firmware change)	100 Hz
u-blox 6T	High Sensitivity, Timing	L1 GPS	5 Hz
SiRF IV	High Sensitivity	L1 GPS	1 Hz
Teseo II	High Sensitivity GNSS	L1 GPS/GLONASS	1 Hz

**Table 2.** Non GNSS NavCube sensors included on internal carrier board.

**Sensor**	**Sensor Application**	**Key Parameters**	**Update Rate**
ADIS16375	State of the art < $1,000 MEMS IMU	Gyros: In Run Bias Stability: 12 °/hr (1) Dynamic Range: ±350 °/s (typ.) Accelerometers: In Run Bias Stability: 0.13 mg (1) Dynamic Range: ±18 g (min.)	100 Hz
HMC5883L	Embedded compassing	2 mg resolution 3 axis measurement	100 Hz
BMP085	Embedded altimeter	3 Pa RMS noise	25 Hz

**Table 3.** NavCube Power Usage by Component Type.

**Component**	**Voltage Rail Utilized (V)**	**Net Usage (W)**
OEM6 − Digital + Antenna	3.3	1.8
File System	3.3	0.33
SiRF IV	3.3	0.10
u-blox 6T	3.3	0.15
Graphical LCD	5.0	0.85
Core Controller	3.3	0.35
Serial Port Replicators	3.3	0.45
Serial Level Shifters	3.3	0.35
Indicator LEDs	3.3	0.2
XBee Link	3.3	1.0
Adaptable Sensor Pod (Internal)	5.0	1.0
Adaptable Sensor Pod (External)	5.0	1.0/unit

**Total System Power**	**7.58 W or 9.3 h**	

**Table 4.** NavCube Sensor Data Rates.

**Sensor**	**Approximate Data Rate**		**Maximum Observation Frequency (Hz)**
	**KB/s**	**MB/Hour**	
Novatel OEM6	60	211	100
ASP (Int. or Ext.)	6.0	21	100
u-blox 6T	4.5	16	5
Sirf IV	3.0	11	1
Teseo II	2.5	9	1
XBee Link	11	39	N/A
1 External Sensor	6.0	21	100

**TOTAL**	**93**	**328**	

## References

[1] Kwakkel S.P., Lachapelle G., Cannon M.E. GNSS aided *in situ* human lower limb kinematics during running.

[2] Abdulrahim K., Hide C., Moore T., Hill C. (2012). Using constraints for shoe mounted indoor pedestrian navigation. J. Navig. R. Inst. Navig.

[3] Girard G., Côté S., Zlatanova S., Barette Y., St-Pierre J., van Oosterom P. (2011). Indoor pedestrian navigation using foot-mounted IMU and portable ultrasound range sensors. Sensors.

[4] Jimenez A.R., Seco F., Zampella F., Prieto J.C., Guevara J. (2011). PDR with a foot-mounted IMU and ramp detection. Sensors.

[5] Fuchs C., Aschenbruck N., Martini P., Wieneke M. (2011). Indoor tracking for mission critical scenarios: A survey. Pervasive Mobile Comput.

[6] Park S.K., Suh Y.S. (2010). A zero velocity detection algorithm using inertial sensors for pedestrian navigation systems. Sensors.

[7] Chen R., Chen W., Chen X., Zhang X., Chen Y. (2010). Sensing strides using EMG signal for pedestrian navigation. GPS Solut.

[8] Huang C., Liao Z., Zhao L. (2010). Synergism of INS and PDR in self-contained pedestrian tracking with a miniature sensor module. IEEE Sens. J.

[9] Torres-Solis J., Chau T. (2010). Wearable indoor pedestrian dead reckoning system. Pervasive Mobile Comput.

[10] Skog I., Handel P., Nilsson J., Rantakokko J. (2010). Zero-velocity detection—An algorithm evaluation. IEEE Trans. Biomed. Eng.

[11] Sun Z., Mao X., Tian W., Zhang X. (2009). Activity classification and dead reckoning for pedestrian navigation with wearable sensors. Meas. Sci. Technol.

[12] Godha S., Lachapelle G. (2008). Foot mounted inertial system for pedestrian navigation. Meas. Sci. Technol.

[13] Tan H., Wilson A.M., Lowe J. (2008). Measurement of stride parameters using a wearable GPS and inertial measurement unit. J. Biomech.

[14] Ojeda L., Borenstein J. (2007). Non-GPS navigation for security personnel and first responders. J. Navig. R. Inst. Navig.

[15] Cho S.Y., Park C.G. (2006). MEMS based pedestrian navigation system. J. Navig. R. Inst. Navig.

[16] Foxlin E. (2005). Pedestrian tracking with shoe-mounted inertial sensors. IEEE Comput. Graph. Appl.

[17] Sabatini A.M. (2011). Estimating three-dimensional orientation of human body parts by inertial/magnetic sensing. Sensors.

[18] Liu Z., Aduba C., Won C.H. (2011). In-plane dead reckoning with knee and waist attached gyroscopes. Measurement.

[19] Ayrulu-Erdem B., Barshan B. (2011). Leg motion classification with artificial neural networks usingwavelet-based features of gyroscope signals. Sensors.

[20] Luinge H.J., Veltink P.H. (2005). Measuring orientation of human body segments using miniature gyroscopes and accelerometers. Med. Biol. Eng. Comput.

[21] Terrier P., Schutz Y. (2005). How useful is satellite positioning system (GPS) to track gait parameters? A review. J. NeuroEngineering Rehabilit.

[22] Faulkner W.T., Alwood R., Taylor D.W.A., Bohlin J. GPS-denied pedestrian tracking in indoor environments using an imu and magnetic compass.

[23] Li D., Landry R.J., Lavoie O. Low-cost MEMS sensor-based attitude determination system by integration of magnetometers and GPS: A real-data test and performance evaluation.

[24] Ladetto Q., Merminod B. Digital magnetic compass and gyroscope integration for pedestrian navigation.

[25] Garmin (2012). Forerunner 310XT.

[26] Zhou H.Y., Hu H.S. (2004). A Survey—Human Movement Tracking and Stroke Rehabilitation.

[27] Sato T., Nakamura S., Terabayashi K., Sugimoto M., Hashizume H. Design and implementation of a robust and real-time ultrasonic motion-capture system.

[28] Incorporated, P.T. High Performance Real-Time 3D motion Capture System for Professionals.

[29] (2012). Optotrak Certus Motion Capture System.

[30] Moeslund T.B., Granum E. (2001). A survey of computer vision-based human motion capture. Comput. Vis. Image Underst.

[31] (2012). Six-Axis (Gyro + Accelerometer) MEMS MotionTracking™ Devices.

[32] (2010). ADIS16375 Data Sheet (Rev. A).

[33] (2010). iNEMO: iNErtial Module V2 Demonstration Board Based on MEMS Sensors and the STM32F103RE.

[34] (2010). MTx—3DOF Orientation Tracker.

[35] Bancroft J.B., Lachapelle G. (2011). Data fusion algorithms for multiple inertial measurement unit. Sensors.

[36] Yuksel Y., El-Sheimy N. (2011). An optimal sensor fusion method for skew-redundant inertial measurement units. J. Appl. Geod.

[37] Susi M., Renaudin V., Lachapelle G. Detection of quasi-static instants from handheld MEMS devices.

[38] Bancroft J.B., Afzal M.H., Lachapelle G. High performance GNSS augmented pedestrian navigation in signal degraded environments.

[39] Bancroft J.B. (2010). Multiple Inertial Measurement Unit Integration for Pedestrian Navigation.

[40] Afzal H., Renaudin V., Lachapelle G. (2011). Use of earth’s magnetic field for mitigating gyroscope errors regardless of magnetic perturbation. Sensors.

[41] Renaudin V., Afzal H., Lachapelle G. (2010). Complete tri-axis magnetometer calibration in the magnetic domain. J. Sens. Hindawi.

[42] Waegli A., Skaloud J. (2009). Optimization of two GPS/MEMS-IMU integration strategies with application to sports. GPS Solut.

[43] Lachapelle G., Morrison A., Ong R., Cole G. (2009). A high performance GNSS-based sensor for elite skier training. GPS World.

[44] Waegli A., Guerrier S., Skaloud J. Redundant MEMS-IMU integrated with GPS for performance assessment in sports.

[45] Kwakkel S.P. (2008). Human Lower Limb Kinematics Using GPS/INS.

[46] Zhang K., Deakin R., Grenfell R., Li Y., Zhang J., Camerson W.N., Silcock D.M. (2004). GNSS for sports—Sailing and rowing perspectives. J. Glob. Position. Syst.

[47] Renaudin V., Afzal H., Lachapelle G. New method for magnetometers based orientation estimation.

